# Psychiatric Status across Body Mass Index in a Mediterranean Spanish Population

**DOI:** 10.1371/journal.pone.0145414

**Published:** 2015-12-18

**Authors:** Mario Gutiérrez-Bedmar, Elena Villalobos Martínez, Antonio García-Rodríguez, Carlos Muñoz-Bravo, Alberto Mariscal

**Affiliations:** 1 Department of Public Health and Psychiatry, University of Málaga, Málaga, Spain; 2 Community Mental Health Service, University Hospital Virgen de la Victoria, Málaga, Spain; Vanderbilt University Medical Center, UNITED STATES

## Abstract

**Background:**

Mental and body weight disorders are among the major global health challenges, and their comorbidity may play an important role in treatment and prevention of both pathologies. A growing number of studies have examined the relationship between psychiatric status and body weight, but our knowledge is still limited.

**Objective:**

The present study aims to investigate the cross-sectional relationships of psychiatric status and body mass index (BMI) in Málaga, a Mediterranean city in the South of Spain.

**Materials and Methods:**

A total of 563 participants were recruited from those who came to his primary care physician, using a systematic random sampling, non-proportional stratified by BMI categories. Structured clinical interviews were used to assess current Axes-I and II mental disorders according to the Diagnostic and Statistical Manual of Mental Disorders, Fourth Edition, Text Revision (DSM-IV-TR). BMI was calculated as weight (Kg) divided by square of height in meters (m^2^). Logistic regression was used to investigate the association between BMI and the presence of any mental disorder. BMI was introduced in the models using restricted cubic splines.

**Results:**

We found that high BMI values were directly associated with mood and adjustment disorders, and low BMI values were directly associated with avoidant and dependent personality disorders (PDs). We observed an inverse relationship between low BMI values and cluster A PDs. There were not significant relationships between anxiety or substance-related disorders and BMI.

**Conclusion:**

Psychiatric status and BMI are related in a Mediterranean Spanish population. A multidisciplinary approach to both pathologies becomes increasingly more necessary.

## Introduction

The association between mental health and body weight cannot be refused based on the current scientific literature[1–4]. Both mental diseases and body weight disorders are among the major global health challenges, and many epidemiological studies have reported a dramatic increase in their prevalence and burden of disease[5–8].

Comorbidity between psychiatric and body weight disorders may play an important role in treatment and prevention of both pathologies. Examples of the above are the changes in symptoms of depression associated to weight loss[9], or the improvement of postoperative outcomes in bariatric surgery candidates by a presurgical psychological screening[10]. So, a multidisciplinary approach to both pathologies may be more effective. This multidisciplinary approach should be based on the knowledge and understanding of the relationship between body weight and psychiatric status.

During the last years, a growing number of studies have examined the association between body mass index (BMI) and psychiatric status. Despite the number of studies about this topic, some issues still remain unclear. One of these issues is related to the assessment of psychiatric status. Association between some psychiatric disorders, like mood or anxiety disorders, and BMI has been profusely studied in the literature[2–4,11–29]. However, only a few studies analyses other mental disorders, and the association between some of them and BMI status is, by now, unknown. For example, to our knowledge, there isn’t published any research about the relationship between adjustment disorders and BMI. Further, a comprehensive assessment of psychiatric status allows adjusting for psychiatric comorbidity since psychiatric disorders are associated with both themselves[30] and body weight[11,31,32]

Another important matter is the measurement of body weight. The majority of studies on BMI and psychiatric status are focused on overweight and obese individuals, and only a few include underweight participants. The non inclusion of these individuals involves a significant loss of information because most studies show a nonlinear relationship between the full range of BMI values and mental diseases[12–14,31,33–36]. In general, these studies show U- or J-shaped relationships, with its minimum (best mental health) at a normal weight value. Methodologically this nonlinearity has to be taking into account, because the inclusion of BMI as a continuous independent variable in linear or logistic regression models may be no suitable.

Finally, there is a lack of studies in some epidemiologically important populations. A Mediterranean-style diet has been associated with both better psychiatric status[37,38] and less incidence of overweight or obesity[39,40]. These associations may influence the relationship between psychiatric status and BMI, so it would be interesting to study this relationship in a Mediterranean population, because these data have not been published before.

The purpose of this study was to evaluate the relationship between BMI and psychiatric status in a Mediterranean Spanish population, covering a wide range of both psychiatric disorders and BMI values.

## Materials and Methods

### Population and Sample

The study population consisted of inhabitants of Málaga (South of Spain) aged between 18 and 65 years, who were assigned to a health center within Málaga Health District. To get a sample from this population, a systematic random sample was drawn. The sample was non-proportional stratified by BMI categories in order to include enough participants in extreme categories of BMI. Between January and December of 2011, a total of 563 participants were recruited from those who came to his primary care physician with a disease not related with body weight or mental health. Inclusion criteria were men or women aged between 18 and 65 years, who accepted to participate in the study and signed the Informed Consent Form. Exclusion criteria were presence of a handicap that prevents giving a reliable answer, history of psychiatric disorder during the last two years, intake of drugs related to weight change, and any change in BMI due to: metabolic or neuroendocrine etiology, genetic malformation syndromes, lipomathosis or lipodystrophy.

The Ethics Committee of the Faculty of Medicine of de University of Málaga approved this study.

### Measures

An unique psychiatrist conducted structured clinical interviews to all participants to diagnose current mental disorders according to Diagnostic and Statistical Manual of Mental Disorders, Fourth Edition, Text Revision (DSM-IV-TR)[41]. Concerning Axis I psychiatric disorders, our study examined: schizophrenia and other psychotic disorders, mood, anxiety, adjustment, somatoform, factitious, dissociative, sexual and gender identity, substance use and eating disorders. Within eating disorders, we considered anorexia nervosa, bulimia nervosa, binge eating disorder and eating disorders not otherwise specified (EDNOS). Furthermore, we evaluated ten Axis II personality disorders (PDs): paranoid, schizoid, schizotypal, antisocial, borderline, histrionic, narcissistic, avoidant, dependent, and obsessive-compulsive personality disorder. To ensure a sufficient number of events per variable, we analyzed cluster A (paranoid, schizoid and schizotypal), cluster B (antisocial, borderline, histrionic and narcissistic), and cluster C (avoidant, dependent and obsessive-compulsive), rather than individual PDs. A participant with one or more PDs in a given cluster was considered into this cluster.

Height and weight measures were obtained with the same instruments by trained study staff. BMI was calculated as weight (Kg) divided by square of height in meters (m^2^). According to WHO international classification[42], participants were classified into one of four groups: Underweight (BMI <18.5 kg/m^2^), Normal weight (BMI 18.5–24.99 Kg/m^2^), Overweight (BMI 25.0–29.99 Kg/m^2^) and Obesity (BMI ≥ 30 Kg/m^2^). We used this categorization of BMI only for descriptive analyses, and we used continuous BMI to investigate associations between DSM-IV-TR Axes I and II mental disorders and BMI.

We collected additional information regarding participants’ age (years), gender, educational level (no studies, primary, secondary, university), having a paid work (yes/no), living alone (yes/no), origin (rural/urban), family history of obesity (yes/no), and family history of psychiatric disease (yes/no). Participants were considered with family history of psychiatric disease if their parents or siblings have been diagnosed with psychiatric disorders or received psychiatric treatment four or more times in the last five years. These variables were used to adjust for the possible confounding effect.

### Data Analysis

We described the sample by studying variables according to BMI categories, Axis I and Axis II disorders. Association between categorical variables was assessed using the Chi-square test, or Fisher exact test if they were dichotomous. Association between age and BMI categories was assessed using ANOVA.

Logistic regression was used to investigate the association between BMI and the presence of any mental disorder. BMI was introduced in the models using restricted cubic splines, allowing us to examine non-linear relationships between BMI and the outcome variables. Before data analysis, and based on literature recommendations about location and number of knots[43], we decided modeling BMI using 4 knots placed at the 5^th^, 35^th^ 65^th^ and 95^th^ percentiles of the sample BMI distribution (corresponding with knots at BMI values of 17.9, 24.1, 29.1 and 43.6). Multivariate models were age and gender adjusted, and included any variable whose statistical association with the outcome variable had a p-value <0.25 (Chi-square test)[44]. As reference value of BMI we used 22.5 Kg/m^2^.

All the analyses were conducted with Stata 13.0 (StataCorp, College Station, TX, USA). We used ‘xbrcspline’ command to tabulate and plot odds ratios (ORs) and their confidence intervals (CIs)[45]. The significance level was set at p <0.05 and all CIs were estimated with a confidence level of 95%.

## Results

### Socio-Demographic Characteristics

Of the total sample (*N* = 563), 78 (13.9%) were underweight, 142 (25.2%) had a normal weight, 170 (30.2%) had overweight and 173 (30.7%) were obese. Table 1 shows the distribution of socio-demographic variables and their association with BMI categories. We found statistical association with age, education level, paid work, family history of obesity and family history of psychiatric disease.

**Table 1:** Socio-demographic variables and their association with BMI.

	Total (n = 563)	Underweight (n = 78)	Normal weight (n = 142)	Overweight (n = 170)	Obese (n = 173)	*p* ^1^
**Age (years)**						<0.001 ^2^
mean (sd)	38.8 (12.2)	23.9 (9.5)	40.2 (10.4)	39.3 (12.1)	43.9 (9.3)	
**Gender n(%)**						0.523
Female	328 (58.3)	41 (52.6)	79 (55.6)	103 (60.6)	105 (60.7)	
**Education n(%)** ^3^						<0.001
No studies or Primary	257 (50.8)	35 (44.9)	49 (40.2)	71 (44.1)	102 (70.3)	
Secondary or more	249 (49.2)	43 (55.1)	73 (59.8)	90 (55.9)	43 (29.7)	
**Origin n(%)**						0.069
Rural	74 (13.1)	6 (7.7)	16 (11.3)	20 (11.8)	32 (18.5)	
Urban	489 (86.9)	72 (92.3)	126 (88.7)	150 (88.2)	141 (81.5)	
**Paid work n(%)**						<0.001
Yes	235 (41.7)	13 (16.7)	85 (59.9)	91 (53.5)	46 (26.6)	
**Live alone n(%)**						0.056
Yes	79 (14.0)	11 (14.1)	11 (7.7)	25 (14.7)	32 (18.5)	
**FH** ^4^ **Obesity n(%)**						<0.001
Yes	84 (14.9)	12 (15.4)	7 (4.9)	25 (14.7)	40 (23.1)	
**FH** ^4^ **Psychiatric Disease n(%)**						<0.001
Yes	98 (17.4)	13 (16.7)	13 (9.2)	23 (13.5)	49 (28.3)	

^1^ Chi-square test;

^2^ ANOVA;

^3^ Missing for 57 participants (20 normal weight, 9 overweight and 28 obese);

^4^ FH = Family History of

Concerning age, we found that the higher BMI, the higher age. Pearson’s coefficient correlation between both variables (considered as continuous) was *r* = 0.383 (*p* < 0.001). Educational level differs among groups. We have found a high percentage of people without studies in the obese group and none in the underweight group. Regarding paid work, there are no statistical differences between the normal weight and overweight group, and between underweight and obese group. The presence of family history of obesity is highest in the obese group and lowest in the normal weight group. When we compared the underweight with the overweight group, we found no statistical differences. We found a similar association between weight status and family history of psychiatric disease. The obese group had the highest percentage of family history of psychiatric disease, and the normal weight group had the lowest percentage. Again, when we compared the underweight with the overweight group, we found no statistical differences.

### Axis I Disorders and BMI Status

Table 2 describes Axis I psychiatric disorders by socio-demographic variables and BMI categories. In order to show only relevant information, we took into account only Axis I disorders with enough number of cases. Specifically, we didn’t find any participant with factitious, dissociative, sexual and gender identity or schizophrenia and other psychotic disorders. Further, only one participant was diagnosed with a somatoform disorder. Therefore, we didn’t show these disorders in the table.

**Table 2:** Frequency of Axis I psychiatric disorders by socio-demographic variables and BMI.

	Mood Disorders (n = 43)		Anxiety Disorders (n = 48)		Adjustment Disorders (n = 43)		Eating Disorders (n = 89)		Substance-related Disorders (n = 36)	
	n	(%)	n	(%)	n	(%)	n	(%)	n	(%)
**Gender**										
Male	17	(7.2)	19	(8.1)	19	(8.1)	32	(13.6)	15	(6.4)
Female	26	(7.9)	29	(8.8)	24	(7.3)	57	(17.4)	21	(6.4)
**Age**										
15–24	9	(11.4)	9	(11.4)	3	(3.8)	16	(20.3)	2	(2.5)
25–34	8	(7.0)	12	(10.4)	7	(6.1)	16	(13.9)	6	(5.2)
35–44	17	(8.9)	16	(8.4)	19	(10.0)	30	(15.7)	12	(6.3)
45–54	9	(7.8)	10	(8.7)	8	(7.0)	14	(12.2)	12	(10.4)
55–64	0	0	1	(1.6)	6	(9.7)	13	(21.0)	4	(6.5)
**Education** ^1^										
No studies	10	(13.5)	4	(5.4)	**11**	**(14.9)** *	**26**	**(35.1)** **	7	(9.5)
Primary	15	(8.2)	19	(10.4)	**9**	**(4.9)** *	**27**	**(14.8)** **	10	(5.5)
Secondary	11	(6.8)	16	(9.9)	**14**	**(8.6)** *	**21**	**(13.0)** **	9	(5.6)
University	6	(6.9)	6	(6.9)	**3**	**(3.5)** *	**5**	**(5.8)** **	4	(4.6)
**Paid work**										
Yes	14	(6.0)	15	(6.4)	17	(7.2)	**20**	**(8.5)** **	11	(4.7)
No	29	(8.8)	33	(10.1)	26	(7.9)	**69**	**(21.0)** **	25	(7.6)
**Live alone**										
Yes	4	(5.1)	3	(3.8)	10	(12.7)	12	(15.2)	6	(7.6)
No	39	(8.1)	45	(9.3)	33	(6.8)	77	(15.9)	30	(6.2)
**Origin**										
Rural	**10**	**(13.5)** *	2	(2.7)	6	(8.1)	14	(18.9)	7	(9.5)
Urban	**33**	**(6.8)** *	46	(9.4)	37	(7.6)	75	(15.3)	29	(5.9)
**FH** ^2^ **Obesity**										
Yes	7	(8.3)	11	(13.1)	11	(13.1)	**26**	**(31.0)** **	**13**	**(15.5)** **
No	36	(7.5)	37	(7.7)	32	(6.7)	**63**	**(13.2)** **	**23**	**(4.8)** **
**FH** ^2^ **Psychiatric Disease**										
Yes	12	(12.2)	13	(13.3)	**13**	**(13.3)** *	**29**	**(29.6)** **	**15**	**(15.3)** **
No	31	(6.7)	35	(7.5)	**30**	**(6.5)** *	**60**	**(12.9)** **	**21**	**(4.5)** **
**Body Mass Index** ^3^										
Underweight	**8**	**(10.3)** **	**11**	**(14.1)** *	**2**	**(2.6)** **	**14**	**(18.0)** **	3	(3.9)
Normal weight	**0**	**(0)** **	**8**	**(5.6)** *	**3**	**(2.1)** **	**5**	**(3.5)** **	5	(3.5)
Overweight	**15**	**(8.8)** **	**7**	**(4.1)** *	**10**	**(5.9)** **	**14**	**(8.2)** **	10	(5.9)
Obese	**20**	**(11.6)** **	**22**	**(12.7)** *	**28**	**(16.2)** **	**56**	**(32.4)** **	18	(10.4)

*p<0.05 (Chi-square test);

**p<0.001 (Chi-square test);

^1^ Missing for 57 participants (20 normal weight, 9 overweight and 28 obese);

^2^ FH = Family History of;

^3^ Participants were classified as Underweight (BMI <18.5 Kg/m^2^), Normal weight (BMI 18.5–24.99 Kg/m^2^), Overweight (BMI 25.0–29.99 Kg/m^2^) and Obese (BMI ≥30 Kg/m^2^)

Of the total sample, 43 participants (7.6%) were diagnosed with any mood disorder, 48 (8.5%) were diagnosed with any anxiety disorder, 43 (7.6%) were diagnosed with adjustment disorder, 89 (15.8%) were diagnosed with any eating disorder and 36 (6.4%) were diagnosed with any substance-related disorder. We found more than one Axis I mental disorder in 58 participants (10.3%). From them, 42 had two disorders, 15 had three disorders and only one had four disorders simultaneously.

Among participants with eating disorders, 89 patients, we found 3 cases (3.4%) of anorexia nervosa, 15 cases (16.9%) of bulimia nervosa, 2 participants (2.2%) with EDNOS, 65 (73%) with binge eating disorder and 4 participants (4.5%) with EDNOS and binge eating disorder simultaneously.

Mood disorders (43 patients) were statistically associated with origin, being more frequent in rural areas. We found statistical association between mood disorders and BMI. Higher frequencies of mood disorders were obtained in extreme weight categories (underweight and obese) but we didn’t find statistical differences between underweight and obese groups (p = 0.761). None of the normal weight participants (n = 142) had mood disorders.

Anxiety disorders (48 patients) were statistically associated only with BMI, being more frequent in extreme weight categories. When we compared underweight with obese participants, we didn’t find statistical differences (p = 0.764).

Adjustment disorders (43 patients) were statistically associated with education level (highest percentage in non-studies participants) and family history of psychiatric disease. We found a strong direct relationship between BMI and adjustment disorders.

Eating disorders (89 patients) were inversely associated with education level and paid work, and directly associated with family history of obesity and family history of psychiatric disease. Among BMI categories, we found a high percentage of eating disorders in obese (32.4%) and underweight (18%) participants. When we compared these two categories, we found statistically significant differences (p = 0.018).

Substance-related disorders (36 patients) were statistically associated only with family history of obesity and family history of psychiatric disease.

**Fig 1:**
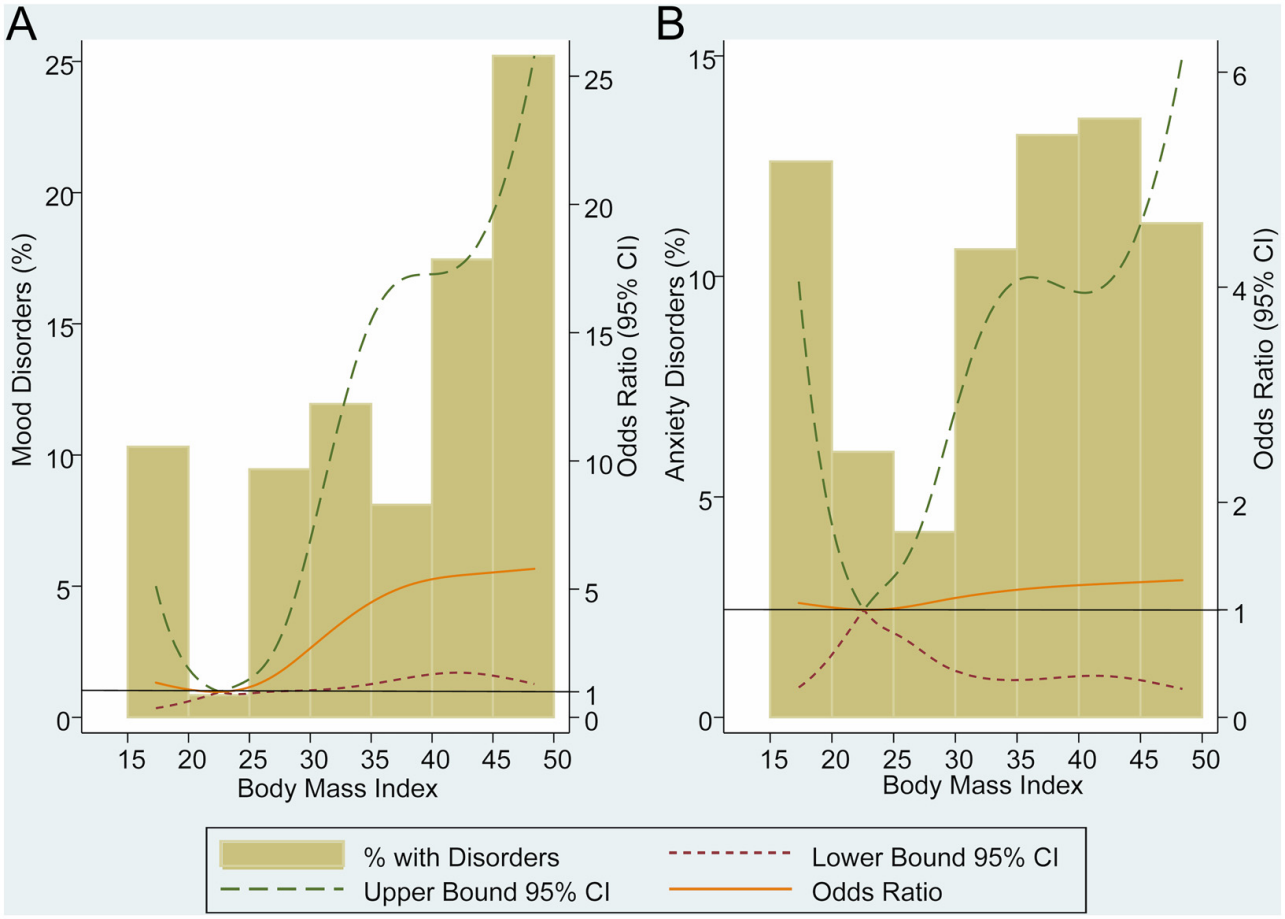
Adjusted odds ratio and percentage of mood and anxiety disorders across the continuum of BMI. (A) Adjusted by age, gender, origin, educational level, family history of psychiatric disease and eating disorders; (B) Adjusted by age, gender, live alone, paid work, origin, family history of obesity, family history of psychiatric disease, substance-related disorders, eating disorder and cluster C personality disorders; CI = confidence interval.

Fig 1 shows the adjusted odds ratio (OR) and percentage of mood and anxiety disorders across the continuum of BMI. ORs were estimated considering a reference level of BMI = 22.5Kg/m^2^. Above BMI reference level we found a direct association between BMI and mood disorders, but below reference level we didn’t find statistical association (Fig 1A). Concerning anxiety disorders and BMI (Fig 1B), we found an U-shaped relationship between rates of anxiety disorders and weight status, but we didn’t obtain any statistically significant adjusted OR.

**Fig 2:**
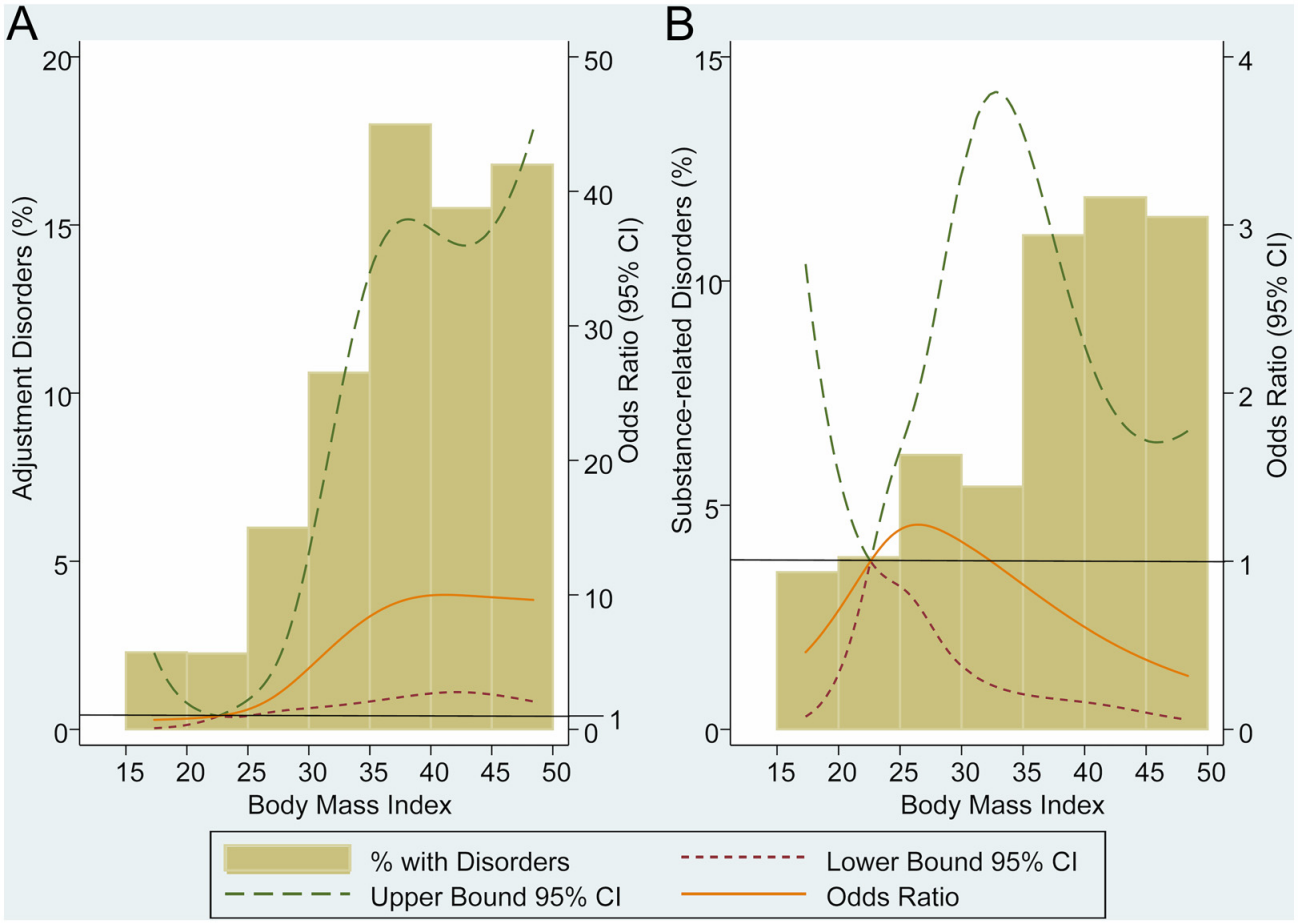
Odds ratio and percentage of adjustment and substance-related disorders across the continuum of BMI. (A) Adjusted by age, gender, origin, educational level, family history of psychiatric disease and eating disorders; (B) Adjusted by age, gender, live alone, paid work, origin, family history of obesity, family history of psychiatric disease, substance-related disorders, eating disorders and cluster C personality disorders; CI = confidence interval.

Relationship between adjustment and substance-related disorders and BMI are shown in Fig 2. As above, these graphs display adjusted OR and percentage of disorders by BMI considered as continuum, and reference level was established at BMI = 22.5Kg/m^2^. We found a positive dose-response relationship between BMI and adjustment disorders (Fig 2A). We didn’t find any statistical association between substance-related disorders and BMI (Fig 2B).

**Fig 3:**
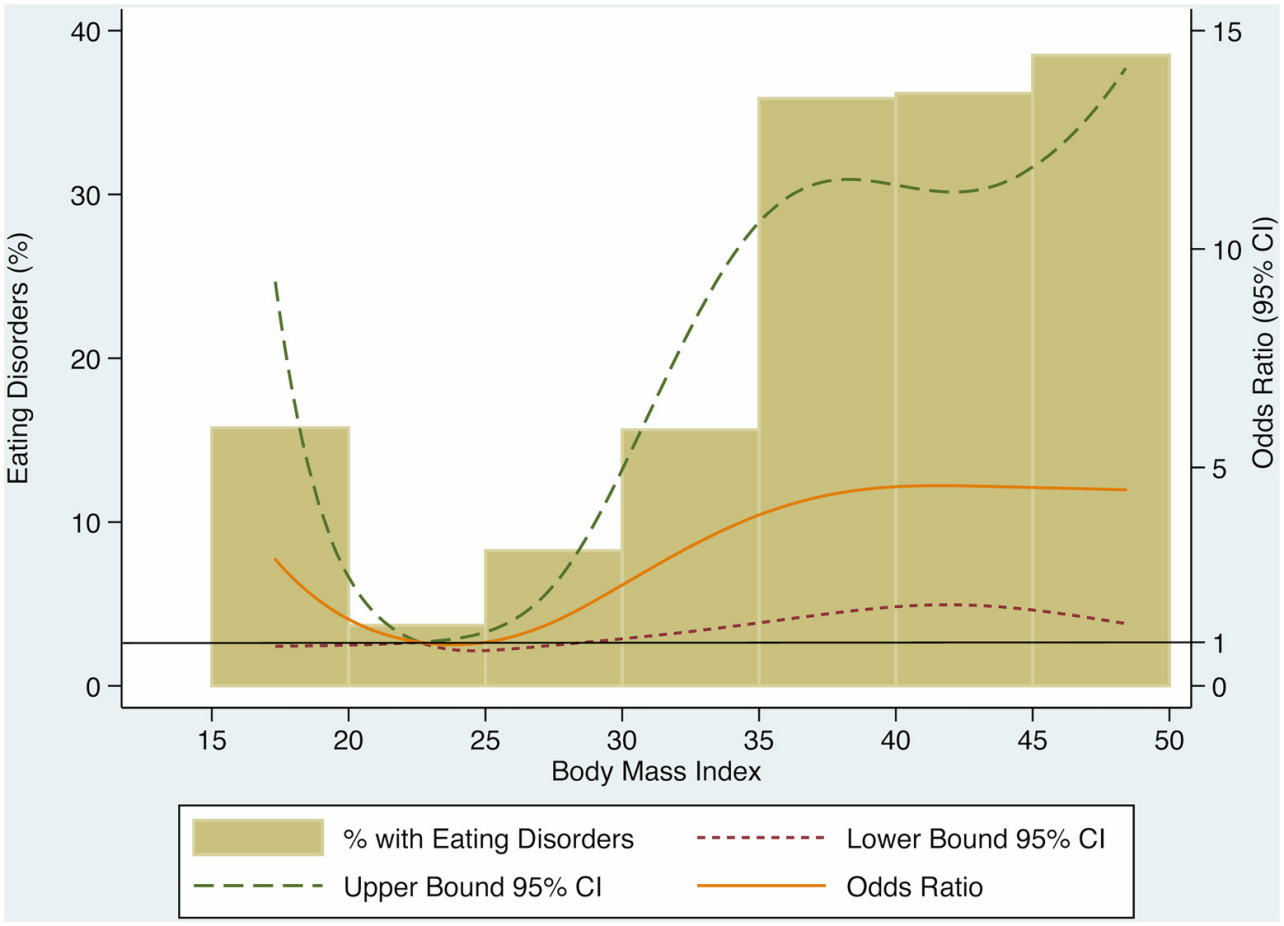
Odds ratio and percentage of eating disorders across the continuum of BMI. Adjusted by age, gender, paid work, educational level, family history of obesity, family history of psychiatric disease, mood disorders, anxiety disorders, adjustment disorders, substance-related disorders, and cluster C personality disorders; CI = confidence Interval.

The last Axis I disorder analyzed was eating disorders. Fig 3 shows its relationship with BMI. The graph displays adjusted OR and percentage of eating disorders by BMI considered as continuum, and reference level was established at BMI = 22.5Kg/m^2^. We found a J-shaped relationship between BMI and eating disorders. Above reference level we found a direct association between BMI and eating disorders, and this relationship was inverse below the reference level. But only in overweight and obese participants the associations observed were statistically significant. The distribution of eating disorders was different among BMI categories. Whereas participants with anorexia nervosa (3 patients) were all underweight, binge eating disorder (65 patients) was present only in overweight and obese participants. We found bulimia nervosa in underweight (n = 11), normal-weight (n = 3) and obese (n = 1) participants.

### Axis II Personality Disorders and BMI Status

Table 3 shows frequencies of any Axis II, cluster A and cluster C PD, by socio-demographic variables and BMI. We found only 7 participants with a cluster B PD, so we didn’t display this cluster in the table in order to show only relevant information.

**Table 3:** Frequency of Axis II disorders by socio-demographic variables and BMI.

	Any Axis II Disorder (n = 101)		Cluster A Disorder (n = 40)		Cluster C Disorder (n = 55)	
	n	(%)	n	(%)	n	(%)
**Gender**						
Male	40	(17.0)	13	(5.5)	26	(11.1)
Female	61	(18.6)	27	(8.2)	29	(8.8)
**Age**						
15–24	16	(20.3)	3	(3.8)	11	(13.9)
25–34	20	(17.4)	5	(4.4)	14	(12.2)
35–44	34	(17.8)	18	(9.4)	16	(8.4)
45–54	19	(16.5)	9	(7.8)	8	(7.0)
55–64	12	(19.4)	5	(8.1)	6	(9.7)
**Education** ^1^						
No studies	19	(25.7)	10	(13.5)	9	(12.2)
Primary	30	(16.4)	10	(5.5)	18	(9.8)
Secondary	26	(16.1)	8	(4.9)	17	(10.5)
University	17	(19.5)	6	(6.9)	9	(10.3)
**Paid work**						
Yes	**30**	**(12.8)** *	**6**	**(2.6)** **	21	(8.9)
No	**71**	**(21.7)** *	**34**	**(10.4)** **	34	(10.4)
**Live alone**						
Yes	16	(20.3)	5	(6.3)	11	(13.9)
No	85	(17.6)	35	(7.2)	44	(9.1)
**Origin**						
Rural	10	(13.5)	3	(4.1)	6	(8.1)
Urban	91	(18.6)	37	(7.6)	49	(10.0)
**FH** ^2^ **Obesity**						
Yes	19	(22.6)	5	(6.0)	13	(15.5)
No	82	(17.1)	35	(7.3)	42	(8.8)
**FH** ^2^ **Psychiatric Disease**						
Yes	24	(24.5)	10	(10.2)	14	(14.3)
No	77	(16.6)	30	(6.5)	41	(8.8)
**Body Mass Index** ^3^						
Underweight	17	(21.8)	**0**	**(0)** *	**14**	**(18.0)** **
Normal weight	16	(11.3)	**12**	**(8.5)** *	**2**	**(1.4)** **
Overweight	30	(17.7)	**11**	**(6.5)** *	**18**	**(10.6)** **
Obese	38	(22.0)	**17**	**(9.8)** *	**21**	**(12.1)** **

*p<0.05 (Chi-square test);

**p<0.001 (Chi-square test);

^1^ Missing for 57 participants (20 normal weight, 9 overweight and 28 obese);

^2^ FH = Family History of;

^3^ Participants were classified as Underweight (BMI <18.5 Kg/m^2^), Normal weight (BMI 18.5–24.99 Kg/m^2^), Overweight (BMI 25.0–29.99 Kg/m^2^) and Obese (BMI ≥30 Kg/m^2^)

Of the total sample, 101 participants (17.9%) were diagnosed with any Axis II PD, 40 (7.1%) were diagnosed with a cluster A PD and 55 (9.8%) were diagnosed with a cluster C PD. We found more than one Axis II PD only in one participant who was diagnosed with both schizotypal and dependent PD.

Among participants with a cluster A PD (40 patients), 9 (22.5%) were diagnosed with paranoid PD, 6 (15%) were diagnosed with schizoid PD, and 25 (62.5%) were diagnosed with schizotypal PD. Regarding cluster C PDs distribution (55 patients), we found 13 (23.6%) participants with avoidant PD, 11 (20%) with dependent PD, and 31 (56.4%) with obsessive-compulsive PD.

As a whole, Axis II PDs (101 patients) were statistically associated only with paid work, being more frequent in participants without paid work. We found the same statistical association between paid work and cluster A PDs.

Concerning BMI we found that, overall, Axis II PDs were not statistically associated with weight status. Nevertheless, when we considered cluster A and cluster C PDs separately, we found a clear statistical relationship between them and BMI. None of underweight participants was diagnosed with a cluster A PD, and when we compared normal weight, overweight and obese group, we didn’t find statistical differences among them (p = 0.525). This relationship was inverted in cluster C PDs. Underweight participants showed the higher percentage of cluster C PDs (18%), and when we compared them with overweight (10.6%) and obese (12.1%) participants, we didn’t find statistical differences among them (p = 0.261).

**Fig 4:**
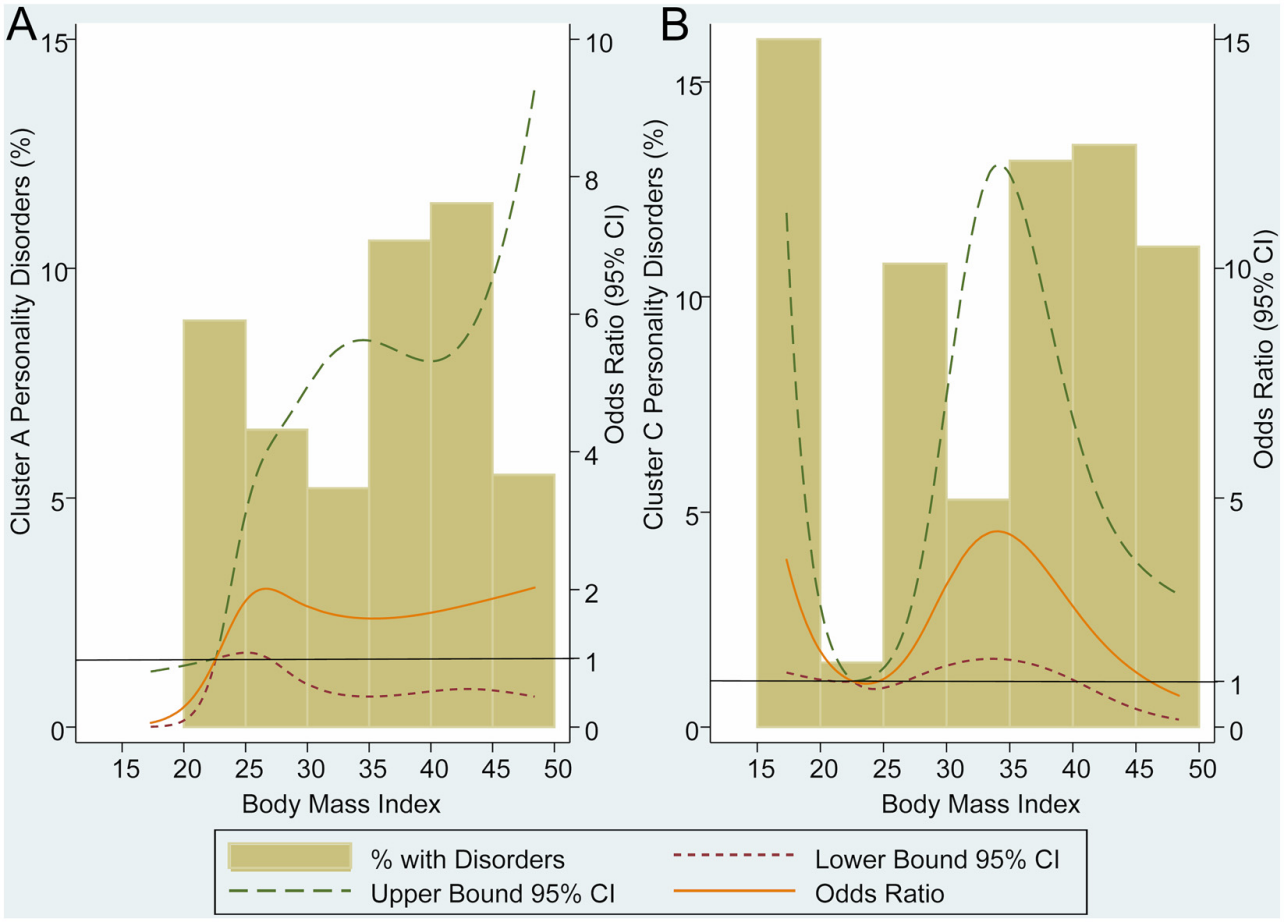
Odds ratio and percentage of cluster A and C personality disorders across the continuum of BMI. (A) Adjusted by age, gender, paid work, educational level, family history of psychiatric disease and substance-related disorders; (B) Adjusted by age, gender, live alone, family history of obesity, family history of psychiatric disease, anxiety and eating disorders; CI = confidence interval.

Fig 4 shows the adjusted ORs and percentage of cluster A and cluster C PDs across the continuum of BMI. ORs were estimated considering a reference level of BMI = 22.5Kg/m^2^. Below reference level there were no participants with a cluster A PD, so we found a statistically significant inverse relationship between being underweight and having a cluster A PD. Above BMI reference level, we didn’t find statistical association between cluster A PDs and BMI (Fig 4A).

Below the reference level, we found that the lower the BMI, the higher the risk of cluster C PDs (Fig 4B). Although more than a half of cluster C PDs diagnosed (55 patients) were obsessive-compulsive PD (31 patients), only one of the 14 underweight participants with a cluster C PD had obsessive-compulsive PD, 8 (57%) were diagnosed with avoidant PD and 5 (36%) with dependent PD.

Above the reference level, we found an inverted U-shaped relationship with the maximum OR located at a BMI between 30 and 35 kg/m^2^.

To further analyze the relationship observed between underweight and cluster C PDs, we studied the associations between each cluster C PD and eating disorders by pairs, using Fisher’s exact test. Obsessive-Compulsive PD was positively associated with binge eating disorder (p = 0.041), but was not associated with anorexia or bulimia nervosa. Avoidant PD was positively associated with anorexia (p = 0.001) and bulimia nervosa (p = 0.004), but was not associated with binge eating disorders. Finally, dependent PD was positively associated with bulimia nervosa (p < 0.001), but was not associated with the others eating disorders.

## Discussion

In this cross-sectional study, we have analyzed the relationship between DSM-IV Axes I and II disorders and BMI. This is the first study to examine this relationship in a Mediterranean population. Overall, our results indicate that high body weight is directly associated with mood and adjustment disorders, and low body weight is directly associated with cluster C PDs (specially avoidant and dependent PDs). Cluster A PDs were inversely associated with low BMI. Relationship between eating disorders and body weight status was J-shaped. All of these relationships are important to keep in mind when designing intervention programs to improve either the body weight or the psychiatric status of patients.

We found that mood disorders are directly associated with high BMI (Fig 1A). Relationship between obesity and mood disorders is by far the most studied in the literature[11,14–20], and the majority of studies have focused on depression[2–4,12,13,21–24]. Our findings are concordant with previous studies since there is enough evidence to accept that mood disorders are directly associated with obesity. Despite the number of studies, reciprocity between mood disorders and body weight is still unclear, and longitudinal studies haven’t proved a clear bidirectional relationship between mood disorders and high BMI[3,4,24–26].

In the multivariate analysis, we didn’t find association between anxiety disorders and BMI. Although rates of anxiety disorders were higher in underweight and obese than in normal weight participants, none of the ORs were statistically significant (Fig 1B). This lack of association between anxiety disorders and weight status is not surprising for us since previous studies about this relationship are controversial. Based on cross-sectional studies of large nationally representative samples from different countries, Mather *et al*.[17], Zhao *et al*.[13], Petry *et al*.[11], Scott *et al*.[14,27] and Barry *et al*.[28] found a direct relationship between anxiety disorders and obesity. On the other hand, cross-sectional population-based studies by Bruffaerts *et al*.[18], McLaren *et al*.[19], Simon *et al*.[29], and two recent longitudinal population-based studies[16,25] didn’t find any association between anxiety disorders and obesity. Three of the above studies[11,13,18] also analyzed the association between underweight and anxiety disorders, and none of them observed a statistically significant relationship.

A novel finding of this study is the relationship observed between adjustment disorders and high BMI (Fig 2A). Compared with others mental disorders, there are few epidemiological studies focused on adjustment disorders[46], so this outcome increases the limited epidemiological knowledge about this disease. The association observed between adjustment disorders and high BMI may be explained through the relationship between stress and body weight. A recent longitudinal study about body weight gain and stress in a national, population-based sample of Australians[47] showed that stressful life events that had occurred in the preceding 12 months were positively associated with weight gain but not weight loss. Adjustment disorder cannot be diagnosed in the absence of a stressful event, and the onset of symptoms must be within 3 months of exposure to the stressor[48]. So, a stressful event may originate both an adjustment disorder and BMI gain. It is important to note that the current finding is novel and requires future replication for confirmation.

Substance-related disorders were not associated with BMI. Although not statistically significant, we observed an inverse relationship between substance-related disorders and BMI at high body weight values (Fig 2B). This protective role of obesity against substance-related disorders has been observed in both, longitudinal[16] and cross-sectional[17,18,29,32] population-based studies. There are population-based studies, however, that didn’t find any relationship between substance-related disorders and BMI[14,27,49], and even studies like Petry *et al*.[11] that found an inverse relationship between nicotine dependence and high BMI and a direct relationship between alcohol-related disorders and high BMI.

Relationship between eating disorders and BMI is well known in the literature, and their clinical definition link them with body weight[48]. In our sample, individuals with anorexia nervosa displayed low BMI, and the reverse pattern was found for binge eating disorders. These results are consistent with current knowledge[50–52].

Regarding PDs and body weight, our results are not supported by previous studies in other populations. To the best of our knowledge, only four studies assess the relationship between PDs and BMI using nationally representative data[11,31,32,53], but all of these studies are based on data from the National Epidemiologic Survey on Alcohol and Related Conditions (NESARC), so their results are similar. Those studies that include underweight participants[11,31] didn’t find an inverse relationship between cluster A PDs and underweight or a direct relationship between cluster C PDs and underweight. On the contrary, Mather *et al*. [31] observed that underweight women have higher risk of schizoid PD (cluster A) and Petry *et al*. [11] found that avoidant PD (cluster C) was inversely associated with underweight.

Nevertheless, based on the defining characteristics of PDs, our results may be explained. People with cluster A PDs are described as odd or eccentric, have strange cognitions or ideas and have difficulty relating to others. They avoid activities that require personal interactions like sports, and due to their introversion, they don’t mind their body image. These features of people with cluster A PDs may promote weight gain and would explain the inverse relationship observed between cluster A PDs and underweight. This hypothesis may be supported by studies like Rosmond *et al*.[54,55] which found that people with cluster A PDs showed the highest BMI compared with others PDs clusters and controls, in both men and women.

Following a similar reasoning, people with cluster C PDs are habitually anxious, fearful and excessively afraid of feeling out of control. The relationship observed between underweight and cluster C PDs is based mainly on avoidant or dependent PD (13 of the 14 underweight participants diagnosed with a cluster C PD had one of those PDs). People with these PDs share a hypersensitivity to negative evaluation, are easily hurt by criticism or disapproval, and may look for a low body weight to prevent unwanted criticism.

Another possible explanation of the relationship observed between avoidant/dependent PDs and underweight, may be the strong statistical association observed between these PDs and anorexia or bulimia nervosa. These findings are concordant with those of a meta-analysis reported by Bornstein[56] who showed that the PDs most commonly associated with anorexia and bulimia nervosa were avoidant and dependent.

Due to the cross-sectional nature of our study, the clinical implications of our results are limited by the unknown direction of causality between body weight and mental health. If we assume that psychiatric disorders may motivate unhealthy weight (underweight or obesity), physicians should be caution with psychiatric patients in order to avoid unhealthy changes of weight. This assumption also implies that the treatment of unhealthy weight patients should be accompanied by a psychiatric assessment and treatment (if necessary) because this may be the underlying cause of an extreme body weight. Two examples of the above are the psychological screening of bariatric surgery candidates, which optimizes their postoperative outcomes[10], or the association between the absence of psychopathology and weight recovery in females with anorexia nervosa[57]. And *vice versa*, if we suppose that unhealthy body weight may cause psychiatric disorders, physicians should treat abnormal body weight in order to prevent or improve mental disorders in their patients. An example of the above are the changes in symptoms of depression associated to weight loss[9]. In any case, treatment of mental and body weight disorders should not be independent.

Although our study established significant associations between psychiatric disorders and BMI, there were several limitations for this study. First, in order to avoid lack of statistical power, we have not carried out the analysis separately for men and women, so the effect of the gender interaction has not been studied. Second, as we mentioned above, this study was cross-sectional and no conclusions can be drawn regarding etiological or causal relationships. Third, although we have adjusted for socio-demographic covariates, we did not have information on other conditions that may have affected the associations between BMI and psychiatric disorders. For example, our participants had not been diagnosed with a psychiatric disease before their inclusion in the study. Probably, they weren't taking any medication related to mental diseases with side effects as weight gain, but we have not information about it. Fourth, although and experienced psychiatrist made all diagnoses through structured clinical interviews, a single-observer bias could be present in this study. And finally, our participants were recruited from those who came to his primary care physician with a disease not related with body weight or mental health, so they are not representative of the general population.

Despite these limitations, this study has some strengths. First, psychiatric disorders were diagnosed by structured clinical interviews. This procedure improves the accuracy and precision of the measurement. Second, the non-proportional stratified sample allows us to include a sufficient number of participants in all BMI categories to explore the shape of the relationship with enough statistical power. Finally, the wide assessment of participant’s psychiatric status (Axes I and II) offers us a more comprehensive understanding of the relationship between BMI and psychiatric diseases.

## Conclusions

In conclusion, we have investigated the association between DSM-IV Axes I and II disorders and BMI in a Mediterranean population. Our findings suggest that high body weight is directly associated with mood and adjustment disorders, and low body weight is directly associated with avoidant and dependent PDs. Our results also suggest an inverse relationship between cluster A PDs and low body weight. Neither psychiatric nor body weight disorders should be treated separately. Knowledge and understanding of their comorbidity may help clinicians to provide the best intervention for their patients, and a multidisciplinary approach to both pathologies becomes increasingly more necessary.

## Supporting Information

S1 File Dataset underlying the findings in the study. (XLSX)
